# A Novel Strategy for Extracting Richer Semantic Information Based on Fault Detection in Power Transmission Lines

**DOI:** 10.3390/e25091333

**Published:** 2023-09-14

**Authors:** Shuxia Yan, Junhuan Li, Jiachen Wang, Gaohua Liu, Anhai Ai, Rui Liu

**Affiliations:** 1 School of Electronics and Information Engineering, Tiangong University, Tianjin 300387, China; 2 College of Mechanical and Electronic Engineering, Northwest A&F University, Xianyang 712100, China; wangjiachen0909@163.com; 3 School of Electrical and Information Engineering, Tianjin University, Tianjin 300072, China; 14785857223@163.com; 4 School of Software, Tiangong University, Tianjin 300387, China

**Keywords:** defect detection, semantic information, transfer learning, channel attention

## Abstract

With the development of the smart grid, the traditional defect detection methods in transmission lines are gradually shifted to the combination of robots or drones and deep learning technology to realize the automatic detection of defects, avoiding the risks and computational costs of manual detection. Lightweight embedded devices such as drones and robots belong to small devices with limited computational resources, while deep learning mostly relies on deep neural networks with huge computational resources. And semantic features of deep networks are richer, which are also critical for accurately classifying morphologically similar defects for detection, helping to identify differences and classify transmission line components. Therefore, we propose a method to obtain advanced semantic features even in shallow networks. Combined with transfer learning, we change the image features (e.g., position and edge connectivity) under self-supervised learning during pre-training. This allows the pre-trained model to learn potential semantic feature representations rather than relying on low-level features. The pre-trained model then directs a shallow network to extract rich semantic features for downstream tasks. In addition, we introduce a category semantic fusion module (CSFM) to enhance feature fusion by utilizing channel attention to capture global and local information lost during compression and extraction. This module helps to obtain more category semantic information. Our experiments on a self-created transmission line defect dataset show the superiority of modifying low-level image information during pre-training when adjusting the number of network layers and embedding of the CSFM. The strategy demonstrates generalization on the publicly available PASCAL VOC dataset. Finally, compared with state-of-the-art methods on the synthetic fog insulator dataset (SFID), the strategy achieves comparable performance with much smaller network depths.

## 1. Introduction

**Figure 1:**
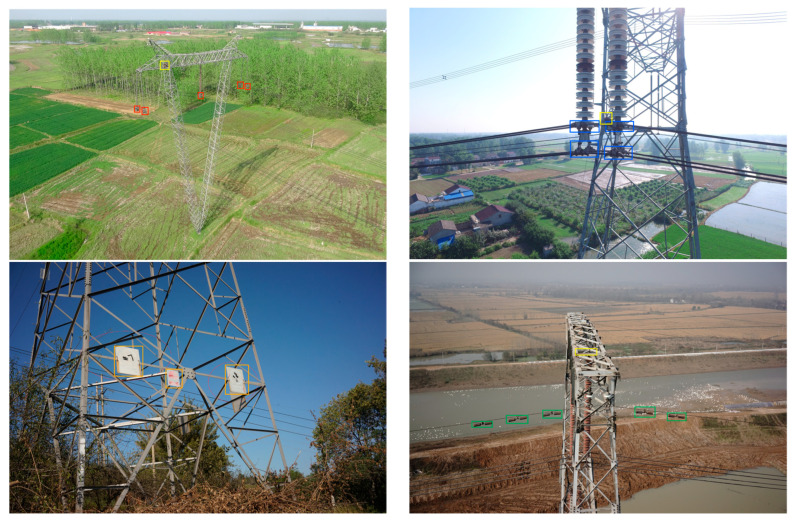
Illustration of power transmission line components. Different colored boxes mark different categories: the yellow marks nests, the blue marks cable clamps, the red marks heavy hammers, the green marks anti-vibration hammers, and the orange marks ancillary facilities.

The overhead transmission line consists of components such as insulators, dampers, triple plates, fixtures, heavy hammers, and tower plates, as shown in Figure 1. As overhead transmission lines are built in different areas of the field, they are often exposed to different environments and climates. The causes of transmission line failures include (1) wire breaks and component wet leakage flashovers caused by extreme weather such as high temperatures, wind and rain, and lightning, (2) short circuits or breakdowns caused by external disturbances such as flying birds and trees, (3) line interruptions due to the aging and corrosion of components or equipment, (4) overloading or transient events (e.g., surges, over-voltages, etc.) caused by uneven electricity consumption behavior or imbalances in the power supply network, (5) ground faults, and (6) human error in operation, construction errors, and theft of electricity. If these problems are not dealt with in time, they can easily cause major safety accidents [1,2], such as large-scale power outages and line damages, which will adversely affect the stable operation of the power system and cause serious economic losses to society [3,4]. Therefore, we need to inspect transmission lines regularly. The complex background and lighting conditions outdoors, small defect sizes, and easily confused categories pose challenges for accurate defect detection. Traditional manual inspection methods of transmission lines, such as using binoculars or climbing towers for close-up views [5], are accurate but costly and pose safety risks [6]. Automatic visual detection of defects in transmission lines is becoming a trend in smart grid development [6,7,8].

Semantic information is crucial for achieving accurate classification tasks in computer vision [9]. Deep networks can capture more abstract and semantic representations of features, making them easier to exploit and understand by machine learning algorithms compared to low-level features like edges and textures. While current approaches often focus on enriching semantic information in deeper networks through increasing depth or multi-scale fusion [9,10,11], there is a need to extract semantic information from shallow networks as well. Furthermore, some scenarios involve small networks and limited computational resources, restricting the depth and availability of high-level semantic information. Therefore, there is a need to explore methods that can effectively extract semantic information from shallow networks while considering the resource constraints.

**Figure 2:**
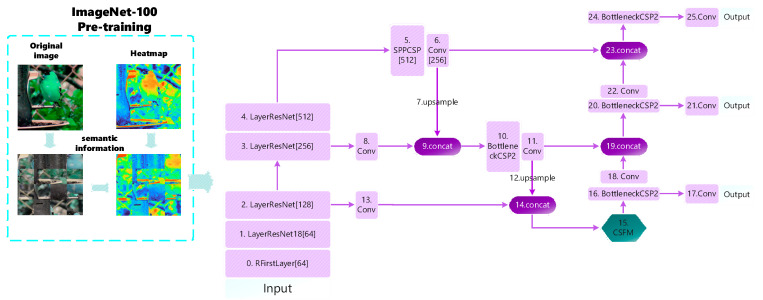
Overall scheme. On the far left is the pre-trained model, which we loaded into the backbone network. ResNet18 is the backbone feature extraction network; neck part FPN and PAN is the feature fusion network. The category semantic fusion module (CSFM) is inserted between the FPN and PAN modules as an articulation between the two. Note that numbers in square brackets such as [64] refer to the number of output channels per module. The heatmaps with different colors represent potential semantic information in images.

Shallow layers have higher resolution and are better suited for processing in the spatial dimension. Inspired by the split of the image in the Vision Transformer (VIT) [12], we attempted a spatial dimensional transformation. The image is segmented into several regions, and then the position of each region is randomly replaced, breaking the image’s fixed features such as position, edge-to-texture connections, etc. In this way, the network’s reliance on these low-level features is reduced, and the focus on semantic information is enhanced. However, if the above operation is performed directly on the image, disrupting the spatial information will inevitably result in the inconsistency between the labeled box and the image, causing a decrease in the training effect, which is verified by our later experimental results. So how can we make the network capable of expressing rich semantic information at an early stage? Again, we tried new ways to make the initial model parameters have this capability. And loading pre-trained models in transfer learning achieves this. We first use the method of image segmentation with a transformed position for pre-training the model of imagenet-100. Potential semantic features of the image are fused into the model parameters, and then the trained model is loaded into a new task. This is used to guide the semantic feature extraction of the shallow network in the new task, as shown on the left side of Figure 2. Our aim in pre-training is not to learn a certain feature in advance but to learn a certain ability: the ability to extract underlying semantic features. If shallow networks are made capable of acquiring this capability, then the network can still acquire advanced semantic features without increasing the depth of the network. In this way, the lightweight network also achieves better detection performance. Since high-level semantic information focuses on categories, attributes, and semantic concepts of objects rather than specific positional information, it is not sensitive to the change in the position of the object in the image. So we try to disrupt the spatial position of the image. Due to the property of the shared parameters of convolutional operations, the same features can be extracted even if the position changes, thus enhancing the position invariance of the high-level semantic features. We use this transformation in pre-training models so that the shallow network can enhance the extraction of semantic information while focusing on texture and edge details.

Since channel attention in the attention mechanism also has the use of category semantic information, we introduced the channel attention mechanism in the feature fusion phase, as shown in Figure 2, No. 15. Channel attention has shown significant advantages in many image processing tasks. For example, in image classification tasks, channel attention can help the network to better distinguish feature differences between different categories [13,14,15,16,17]. In a target detection task, channel attention improves the network’s ability to accurately locate and recognize targets [18,19,20,21,22]. However, current channel attention modules simply and crudely use global pooling in the compression process, which may lose locally important information and lead to relatively limited modeling of complex semantic relationships. We added additional branches based on channel attention to supplement the global information lost in channel attention. This allows important channel information to be obtained while preventing too much information from being lost, and then the detection accuracy is improved by introducing local context normalization [23] to capture the correlation of local features. We also explored the insertion method; the different positions and numbers of insertions will affect the training effect.

We summarize our contributions as follows:

1. Aiming at the challenges of limited computational resources of lightweight embedded devices and easy confusion of defect categories in the field transmission power lines defect detection, we propose a strategy with a lightweight network to acquire high-level semantic features, which is capable of extracting rich semantic feature representations without excessively increasing the depth of the network and improves the detection accuracy of the network. Compared to SOTA, our strategy achieves comparable performance with a small number of network layers.
2. To address the problem of ignoring shallow semantic information, one scheme is proposed to extract shallow semantic information without increasing the depth of the network. The inherent shallow features such as texture and location are broken in the pre-training stage to reduce the network’s dependence on shallow features. And the contrast learning capability of the Simsiame [24] network is utilized to mine the intrinsic semantic feature representations of images. Then, transfer learning is utilized to fine-tune small datasets in practical defect detection to leverage the powerful semantic representations learned from the pre-trained models and guide the extraction of shallow semantic information in the new task.
3. In the feature fusion stage, to obtain more semantic information, we design the category semantic fusion module (CSFM) to focus more on categories. The channel attention is used to extract important channel features and again retain the initial features by one more branch. Also, the association of local features is modeled. Richer semantic information is fused by synthesizing global information and locally important features. This improves the detection accuracy of the network.

This paper is organized as follows: In Section 2, the related work in the detection of defects in power transmission lines, semantic information, transfer learning, channel attention, etc., is generalized. In Section 3, a detailed description of the main methods and principles of the specific structure of the model is given. In Section 4, the experiment, including the experimental environment, the deployment of the experiment, and the analysis of the corresponding experimental results are elaborated. In Section 5, conclusions are finally drawn.

## 2. Related Work

### 2.1. Detection of Defects in Power Transmission Lines

The loading mechanism of power transmission lines mainly involves the power distribution and adjustment of the transmission capacity of the transmission lines. Common methods include load balancing based on load changes, power allocation by changing the series-parallel relationship of transmission lines, and the use of high-voltage transmission and smart grid technology to improve the transmission capacity. Based on the six causes of faults listed in the introduction section, we found the following solutions: (1) use lines and devices designed to resist wind and water, (2) install isolation equipment and regularly trim the surrounding vegetation, (3) conduct regular equipment testing and maintenance and promptly replace aging, damaged, or malfunctioning equipment, (4) regularly monitor and assess the load conditions and install over-voltage protection devices at important nodes and key equipment, (5) enhance the design and maintenance of the grounding system and regularly check the insulation status of cables laid underground, and (6) strengthen the training and awareness of operators and install safety monitoring equipment to monitor the status of the power grid. Common fault detection methods include inspection tours, infrared thermal imaging [25,26], ultrasonic testing [27], and online monitoring systems [28,29]. These methods can help detect and repair abnormal conditions in the transmission line promptly. With the development of deep learning technology, automatic defect detection based on computer vision has been developed. Zhao et al. [30] conducted an in-depth study on the appearance of bolt defects and proposed a bolt form-based nut loss detection method. Manninen et al. [31] established a deep-learning-based equipment defect detection model suitable for transmission line inspection, which can automatically identify and detect common defects in the images. Liu et al. [32] collected sample images of key targets under different backgrounds and lighting conditions to construct a large standard dataset. By optimizing the detection network, the extraction ability of small target features was improved.

### 2.2. Semantic Information

From a probabilistic perspective, semantic information in an image can be measured using information entropy. Information entropy quantifies the uncertainty of a random variable and represents the average amount of information in a given source. In images, semantic information refers to information related to objects, scenes, and concepts. Through computer vision techniques, features and representations can be extracted to describe the probability of occurrence and the relevance of different regions, objects, or scenes in an image. The entropy of this distribution can be used to measure the uncertainty of the semantic information in the image. A higher entropy indicates richer, more diverse, and uncertain semantic information, while a lower entropy indicates more homogeneous and certain semantic information. Semantic information was first often used in the field of natural language processing [33,34] and later applied to the image field and mostly used for semantic segmentation tasks [35,36]. The field of object detection has also noticed the value of semantic information and has begun to utilize semantic information in object detection tasks. The field of object detection mostly utilizes high-level semantic information from the deep network, which is more advantageous for understanding image representations and determining target categories. An essay [37] proposes a deep convolutional neural network model for semantic segmentation of remote sensing images based on multi-scale information fusion. An article [38] increases the target detection scale, refines the feature map, enhances the fusion of deep and shallow semantic information of the feature map, and improves the target detection accuracy. A multilevel structured search-attention fusion network based on multi-layer semantic information guidance is proposed to realize the fusion of infrared and visible images in an end-to-end manner [39].

### 2.3. Transfer Learning

The cost of training deep learning models from scratch is prohibitive for convolutional neural networks (CNNs), which require large amounts of training data for better performance [40,41]. Because training with such a large dataset is a time-consuming process [40], high-performance graphics processing units (GPUs) are also required for fast processing in CNN training. In addition, the training process of CNNs is very complex, and due to convergence and overfitting, the parameters need to be constantly adjusted to ensure equivalent learning of all layers [42]. One approach to solving these problems is transfer learning, helping us utilize knowledge gained from previously constructed models. An article [43] demonstrates that the iris recognition model obtained by performing transfer learning performs better than the model trained by itself. An article [44] proposes a concise and effective knowledge transfer learning strategy called continuous pre-training (CSPT) based on the idea of non-stopping pre-training in natural language processing (NLP), taking into account self-supervised pre-training and a powerful Vision Transformer (ViT) architecture. In this study [45], transfer learning utilizing pre-trained deep convolutional neural networks (ConvNets) is proposed to automatically identify 11 different classes of plasma cells in effusion cytology. Four pre-trained ConvNet architectures were fine-tuned on the plasma cell dataset, i.e., AlexNet, GoogleNet, ResNet, and DenseNet. Similar to the above, we use the method of cutting transformations on images in the pre-training of ImageNet-100 and utilize its learned semantic information parameters to guide the learning of new downstream tasks.

### 2.4. Channel Attention

Attention mechanisms are a potential means of enhancing deep convolutional neural networks. SENet [46] first proposed an effective mechanism for learning channel attention and achieved satisfactory performance. Subsequently, the development of attention modules can be broadly characterized in two directions: (1) enhanced feature aggregation and (2) a combination of spatial and channel attention. Channel attention in CBAM [47] is placed after spatial attention, which uses average pooling and maximum pooling to aggregate features and two fully connected layers to extract channel weights. GSoP [48] introduces second-order pooling for more efficient feature aggregation. GCNet [49] shares similar principles with non-local neural networks. It develops a simplified non-local model and integrates it with the SE block, resulting in a lightweight module for modeling long-term dependencies. Double attention networks [50] introduce a new relation function for non-local blocks for image and video recognition. DANet [51] also considered Non-local-based channel and spatial attention for semantic segmentation. However, most non-local-based attention modules have high model complexity. The above approaches have focused on developing complex attention modules that have better performance. Unlike them, ECA [52] aims to reduce model complexity to learn effective channel attention and proposes a local cross-channel interaction strategy without dimensionality reduction. The strategy can be efficiently implemented by one-dimensional convolution. A method is proposed to adaptively select the kernel size of the 1D convolution to determine the coverage of local cross-channel interactions.

## 3. Methods

The small size and similar morphology of power transmission line defects cause difficulty in classification and separation from complex backgrounds, so we focus on utilizing advanced semantic feature representation. And considering the limited computational resources of lightweight embedded devices, lightweight neural networks are deployed. The overall framework proposed in this paper is shown in Figure 2. The model can be divided into two modules. The first part is a shallow network semantic information extraction scheme. The second part is the design of the CSFM. A more intuitive diagram of the overall structure can be seen in Figure 2.

### 3.1. Shallow Network Semantic Information Extraction Scheme

Lightweight networks have gained attention for object detection due to computational limitations. Since there are not many layers in the lightweight network, the deeper high-level semantic information is not as rich and underutilized. Concerning fault detection on transmission power lines, we focus on the problem of breakage or detachment due to aging and corrosion of wire components or equipment. Due to the small size and similarity of these components, our goal is to accurately categorize these defective originals. Semantic information can provide more intrinsic information for identifying differences in different transmission line components (e.g., damper, triple plate) or other targets (e.g., nest, tower plate), which is helpful in classification. So how to make the network acquire the ability to express rich semantic information at the initial stage is the goal of our research. Inspired by the idea of image slicing in VIT, we intuitively believe that transformations in spatial dimensions, as well as changes to intrinsic low-level features such as edges and textures, can enable the network to learn potential semantic information within the shallowly categorized target. Based on this, we propose two solutions.

**Figure 3:**
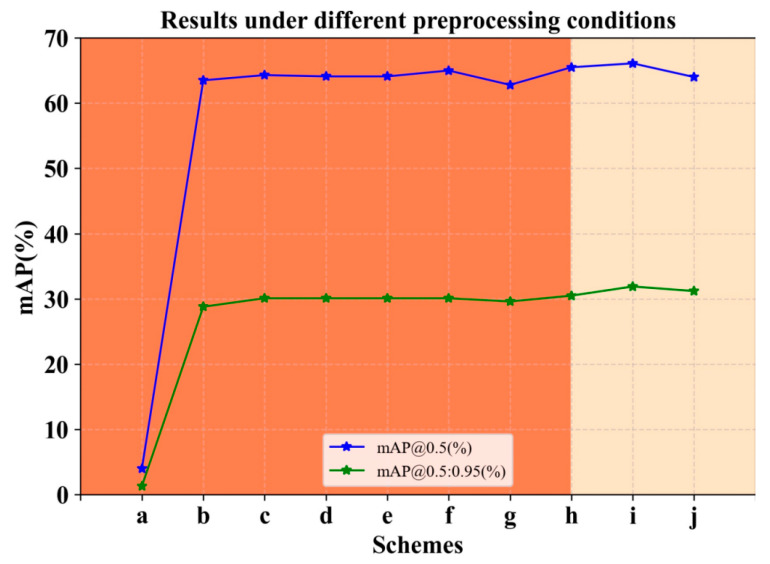
Comparison experiment of different pretreatments. The mAP results of schemes a–j illustrate that direct image segmentation with shifting is the worst (a), pre-training only by dividing the image into 4 blocks for transformation (f) is the best, and pre-training with 16 blocks after fusion of CSFM (i) is the best.

The first scheme is implemented by splitting the image to be trained into several blocks and then replacing the position of each block at random. Disrupting the relative spatial information and texture connections of the image helps to increase the diversity of the data so that the network can better perceive local features and extract more semantic information. By randomly transforming the position of each piece, the shallow network can be made more position-invariant for features at different positions. This means that even if the position of the image block in the image changes, the network is still able to recognize and extract the same semantic information. However, the experimental results are not satisfactory (Figure 3a). We found that directly applying the above processing to the image would cause inconsistency with the position of the labeled boxes, thus interfering with the training process.

Due to the labeled boxes, scheme I cannot be implemented with supervision, so self-supervised learning is a more suitable current task. For the initial network model to have this semantic feature representation, pre-training in transfer learning is employed. And in the pre-training phase, the Simsiame [24] network in contrast learning is deployed. The above method of image segmentation with transformed position is first used to pre-train the ImageNet-100 dataset. Here, the image is segmented into $n=2^{i}$ blocks. During the experiment, we also explored the value of n, where i was set to 2, 3, 4, and 5 for pre-training, and the experimental results can be seen in Figure 3b–g. Based on the final experimental results, for i, it was finally determined that using a value of 4, i.e., dividing the image into 16 pieces, would be the best result. Pre-training of ImageNet-100 in a self-supervised scenario used Simsiame. The pseudo-code of scheme II is in Algorithm 1. To increase readability, keywords are marked in green font and comments are marked in blue font. This is done as follows:
**Algorithm 1** Pseudo code of shallow network semantic information extraction scheme.**  Input:** sequence of image blocks *T* **  Method:** crop_trans: segmentation and random spatial transformation,    rand_aug: random augmentation,    e: feature extractor,    m: mlp prediction header,    S: negative cosine similarity **  Variable:**
*ɑ*: hyperparameter, *n*: number of blocks for image segmentation 1 **import** tensorflow **as** tf 2 **for** *t* **in** *T*: * # load images* 3 *t1*, *t2* = crop_trans(*t, n*), rand_aug(*t*) 4 *f1*, *f2* = e(*t1*), e(*t2*) 5 *p1*, *p2 = m(f1)*, *m(f2)* 6 *loss* = ɑ*S( *p1*, *f2*)+(*1-ɑ*)*S( *p2*, *f1*) 7 8 **def **crop_trans(*t*, *n*) : 9    *blocks* = Lambda(lambda *x*: tf.image.extract_patches(*x*)(*t*) 10  *block_shape* = tf.shape(*block*s) 11  *num_blocks* = *block_shape*[1]*block_shape[2] 12  *blocks* = tf.reshape(*blocks*, [*block_shape*[0], *num_blocks*, *n*, *n*, 3]) 13  *# Random space transformation* 14  **return**
*Tc* = tf.random.shuffle(tf.transpose(*blocks*, perm=[0, 2, 1, 3, 4])) 15 16 **def** S ( *p*, *f* ) : *# negative cosine similarity* 17  *pn* = normalize( *p*, dim=1) * # l2 normlization* 18  *fn* = normalize( *f*, dim=1) * # l2 normlization* 19  **return** - p*f
**/ **pn*fn


The image is first divided into $n=2^{i}$ blocks to obtain the sequence of blocks $T=[T_{1},T_{2},T_{3},\ldots ,T_{n}]$. The segmentation and random spatial transformation operations are then performed to obtain a new sequence of blocks $T_{c}$ (Equation (1)).

(1) $$T_{c}=[random(T_{k})],\;k\in [1,n]$$

The feature extractor is used to perform feature extraction on the image to obtain the feature vector $F_{c}$. A prediction header (MLP network) is added on top of the feature extractor to map the feature vector to a prediction vector $P_{c}$. It is also required to randomly select another augmented sample $T_{m}$ for feature extraction to obtain a feature vector $F_{m}$, which is mapped to a prediction vector $P_{m}$. See Equations (2) and (3).

(2) $$F_{c}=mlp(T_{c})$$

(3) $$F_{m}=mlp(T_{m})$$

The negative cosine similarity of the two samples is calculated (see Formulas (4) and (5)). Finally, the network is trained using the objective function of minimizing negative cosine similarity Equation (6), where α is a hyperparameter used to control the degree of smoothing of the similarity distribution.

(4) $$S^{N}{}_{\left(c,m\right)}=-\frac{P_{c}\cdot F_{m}}{\left\|P_{c}\right\|*\left\|F_{m}\right\|}$$

(5) $$S^{N}{}_{\left(m,c\right)}=-\frac{P_{m}\cdot F_{c}}{\left\|P_{m}\right\|*\left\|F_{c}\right\|}$$

(6) $$Loss=\alpha S^{N}{}_{\left(c,m\right)}+(1-\alpha )S^{N}{}_{\left(m,c\right)}$$

Contrast learning between samples is enhanced by minimizing the differences between samples due to varying spatial, edge features to reduce the shallow network’s over-reliance on low-level features, allowing it to learn potential semantic features of the image. In this way, the pre-trained models are helped to learn richer and more accurate semantic feature representations. The trained model is then loaded into a new task, and the learned semantic feature representations are used to guide the semantic feature extraction of the shallow network in the downstream task.

### 3.2. Category Semantic Fusion Module

The input feature layer is divided into two branches. One branch enters the channel attention module and uses the channel attention to extract important channel features. The other branch only adjusts the image dimensions by simple convolution and retains the initial features. The channel attention here is the channel attention CAM in CBAM. Relative to SENet, it uses not only global average pooling but also global maximum pooling when compressing the image, i.e., it retains the global information and highlights the important features of each channel. Then, the channel features are extracted through two fully connected layers, and each channel gets different weight values indicating different degrees of importance. Then, the channels of the two branches are concatenated. The number of concatenated channels becomes twice the original number, which contains the channels that have been weighted by the channel attention and the channels that have retained the initial features. By combining the information of all channels, important channel features are obtained while avoiding too much global information loss. Global features can provide information about the relationship and context between the power line defect and its surroundings, helping to determine the location and extent of the defect in a complex background. The important channel features obtained allow the network to be more able to discriminate between power line defects with different characteristics, such as easily confused vibration hammers vs. wire clips. After concatenating all the channels, we locally normalize each sample using local context normalization. Unlike global normalization, it is better able to accommodate differences between samples. It emphasizes the local information and details of each sample, complementing the local information that is not attended to by the channel’s attention. The LeakyReLU activation function is then used, which introduces a small slope when the input is negative to retain a portion of the negative information. Enhanced gradient flow helps to extract information characterized by negative values. Finally, the features of all channels are extracted by convolution, which reduces the number of channels to the original number of channels, reduces the computational cost, and also fuses the synthesized feature information.

**Figure 4:**
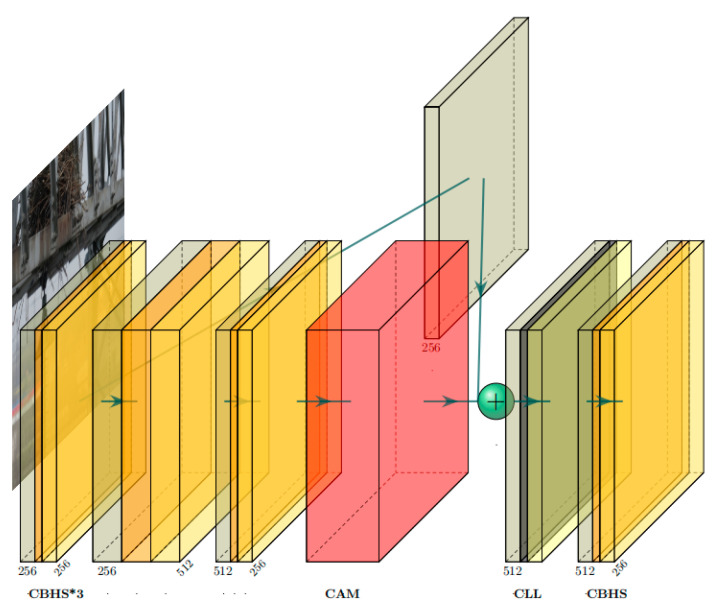
CSFM structure: the CBHS module includes 2D convolution, batch normalization, and hard swish activation function; CAM denotes channel attention module; CLL is 2D convolution, local context normalization, and LeakyRelu activation function.

The details are as follows, as shown in Figure 4:

Supposing that the input feature is $X=[x_{1},x_{2},\ldots ,x_{c}]$ (c is the number of channels), it will go into two separate branches:

Branch 1, the channel attention module, will give each channel a weight value $W=[w_{1},w_{2},\ldots ,w_{c}]$ to output $y_{1}=[x_{1}w_{1},x_{2}w_{2},\ldots ,x_{c}w_{c}]$. Branch 2 retains the initial feature information $y_{2}=X=[x_{1},x_{2},\ldots ,x_{c}]$. Then, the two branches are concatenated as shown in Equation (7).

(7) $$y_{\mathrm{cat}}=[y_{1},y_{2}]=[x_{1}w_{1},x_{2}w_{2},\ldots ,x_{c}w_{c},x_{1},x_{2},\ldots ,x_{c}]$$

Next, the normalization is performed, $y_{lcn}=[y_{1}^{scaled},y_{2}^{scaled},\ldots ,y_{c}^{scaled}]$, where $y_{i}^{scaled}$ is derived from Equation (8), ε is a smaller value to avoid dividing by zero, ${\left[\cdot \right]}_{↓}$ denotes a descending dimension, ${\left[\cdot \right]}_{↑}$ denotes ascending dimension, and $\langle \cdot \rangle$ denotes average pooling. $\gamma_{i}$ and $\beta_{i}$ are the zoom and bias parameters for each channel, respectively.

(8) $$y_{i}^{scaled}=\gamma_{i}{\left[\langle \sqrt{{\left[y_{i}^{2}\right]}_{↓}+\varepsilon }\rangle \right]}_{↑}+\beta_{i},\;(i=1,2,\ldots ,c)$$

The activation function LeakyRelu is referenced in Equation (9), which is implemented based on the pixel level on each feature layer, where an entire feature layer is represented by a pixel. If an entire feature layer is represented in pixels, it is $y_{{}_{i}}^{\prime }=P_{h\times w}^{i}$. Finally, the number of channels is reverted using convolution, $y=[y_{1}^{\prime },y_{{}_{2}}^{\prime },\ldots ,y_{{}_{c}}^{\prime }]$. For the sake of brevity of expression, some of these convolution processes will not be repeated.

(9) $$p_{k}^{i}=\max(\alpha p_{k}^{i},p_{k}^{i}),\;p_{k}^{i}\in P_{h\times w}^{i}$$

## 4. Experiment

### 4.1. Experimental Configuration

In this paper, the experiments use the following environment configuration: python3.7 based on the PyTorch1.4 framework and GPU model NVIDIA GTX1080Ti, a total of four. The operating system is Ubuntu16.04, with the configuration of CUDA10.0, Cudnn7.6.5.

The training parameter settings are shown in Table 1.

### 4.2. Datasets

A total of three datasets are used for the experiments in this paper. The homemade power transmission line dataset, acquired with UAV (unmanned aerial vehicle) aerial photography, contains seven kinds of defects including damper, triple plate, clamp, heavy hammer, damper broken, nest, and tower plate, with a total of 2877 images, of which 2013 are in the training set, and 864 are in the test set. To expand the training samples, multiple forms of data augmentation operations including random rotations, flipping, mixup, mosaic augmentation, etc., are applied to the training set to simulate different shooting lights, angles, and distances. The second dataset is a new synthetic fog insulator dataset (SFID) from the literature [53]. It is constructed by proposing the synthetic fog algorithm to enhance the UPID [54] dataset using random brightness and fog thickness. It contains 13,718 training and test images. Considering the richness and robustness of the samples, we choose the SFID dataset to further validate our proposed method. The final scheme that yields the best results is then validated on the PASCAL VOC dataset as well. The PASCAL VOC dataset is derived from the PASCAL VOC Challenge (The PASCAL Visual Object Classes), a world-class computer vision challenge. The dataset used in the experiments is the 2007 + 2012 version, which contains 20 categories, 16,551 images in the training validation set, and 4952 images in the test set.

### 4.3. Evaluation of Indicators

Each model is evaluated for performance on a validation or test dataset, and the performance measures use various statistics such as accuracy, precision, recall, etc. The statistics chosen are usually specific to the particular application scenario and use case. For each application scenario, it is important to choose a metric that allows for an objective comparison of models. The most common evaluation metric for object detection is the mean average precision, or mAP. It is based on the confusion matrix, as shown in Table 2, and based on counting the number of results for each prediction, the precision and recall are calculated according to Equations (10) and (11). Statistics of precision versus recall at different thresholds are made into P(r) curves, and the area under the curve is average precision (AP) (Equation (12)). Each category has an AP, and the summed average of the APs of all categories is mAP (Equation (13)), where C denotes the number of categories.

(10) $$precision=\frac{TP}{TP+FP}$$

(11) $$recall=\frac{TP}{TP+FN}$$

(12) $$AP=\int_{0}^{1}P(r)dr$$

(13) $$mAP=\frac{\sum_{i=1}^{C}AP_{i}}{C}$$

### 4.4. Experimental Results

#### 4.4.1. Comprehensive Experimental Results

Firstly, we conducted a comparison experiment on the homemade transmission power line dataset as shown in Table 3. ResNet18 was chosen as the backbone network to extract features and FPN and PAN fused features, the training epoch was 100, and other training parameters are shown in Table 1. After experimental exploration, we determined that adding the CSFM between FPN and PAN and using pre-training with segmented images in blocks of 16 gave the best results for the combination of the two, with an improvement in mAP of 2.6 percentage points compared to the original network.

To further validate the effectiveness and generality of our method, we validated it on the public Pascal VOC dataset, and the publicly available SFID, respectively, with a training epoch of 300, and the other training parameters are shown in Table 1. The results are located in Table 4 and Table 5, respectively. From Table 4, we can see that our method is also effective on the Pascal VOC dataset, where mAP@0.5 improves by 0.9 percentage points compared to the original network, while we further compute mAP@0.5:0.95 to find an improvement of 1.6 percentage points.

To show the generality of our proposed strategy, we compare it with state-of-the-art FINet and classic methods. FINet is a specially designed fault detection model. These classical models have also been used for fault detection [55,56,57,58,59]. The comparison experiments on the synthetic fog insulator dataset (SFID) are shown in Table 5, and the results from FINet to YOLOv5 are taken from article [53], where ‘-’ indicates data not mentioned in this article. We set the same epoch (300) for training as the article. We calculated precision and recall for iou = 0.5 and found that our results were better. When the depth (number of layers) of our network is much smaller than that of other state-of-the-art networks, mAP can still match them, thus realizing the goal of high accuracy for lightweight networks. The novelty of our research strategy lies in the fact that we obtain detection results with less network depth (177 layers) which is equivalent to the performance of larger networks (311 layers).

#### 4.4.2. Ablation Experiment

1. Different preprocessing

  To explore the two schemes mentioned in Section 3.1, we conducted the following experiments with ResNet18 as the backbone network, respectively:
  - Randomly initialize and preprocess the image itself by cutting and changing its position, i.e., Scheme 1.
  - Randomly initialize, without any processing of images and networks, as a reference group.
  - In Scheme 2, set the value of i to 5; that is, divide the image into 32 blocks for pre-training.
  - In Scheme 2, set the value of i to 4; that is, divide the image into 16 blocks for pre-training.
  - In Scheme 2, set the value of i to 3; that is, divide the image into 8 blocks for pre-training.
  - In Scheme 2, set the value of i to 2; that is, divide the image into 4 blocks for pre-training.
  - In Scheme 2, set the value of i to 1; that is, divide the image into 2 blocks for pre-training.
  - Based on the c-experiment, the part of the network fusion is inserted into the CSFM.
  - Based on the d-experiment, the part of the network fusion is inserted into the CSFM.
  - Based on the j-experiment, the part of the network fusion is inserted into the CSFM.

The experimental results are shown in Figure 3; the effect of Scheme 1 is the worst. The reason is that the direct processing of the image in the supervised case will destroy the one-to-one correspondence between the labeled box and the image, confusing the labeling, and the network cannot extract the effective features. Comparison experiments for Scheme 2 showed that the highest mAP@0.5 was obtained when pre-training was performed to split the image into 4 blocks for transformations (f), followed by 32 blocks(c) and 16 blocks(d), and the results for mAP @0.5:0.95, c, d, e, and f are equal. So based on pre-training, secondary filtering is implemented and the CSFM is added to the network fusion part to compare the combined results in Figure 3h–j. After comparison, it appears that 16 blocks of pre-training combined with the CSFM works best. Our analysis suggests that the integrated CSFM helps the model to focus on the image regions relevant to the detection task. In the case of a 16-block image, the larger spatial context information already provides enough background information, and the integrated CSFM can help the model better focus on important regions and suppress irrelevant regions, thus improving detection accuracy. Cutting the image into a larger number of chunks can provide a larger range of spatial contextual information as opposed to cutting four chunks. This is particularly important for an integrated CSFM, which can fully utilize the relationships between these blocks to obtain more global contextual features and thus improve detection performance. So we finalized the pre-training program of cutting 16 blocks.

2. An Exploration of Network Depth and Detection Effectiveness

**Figure 5:**
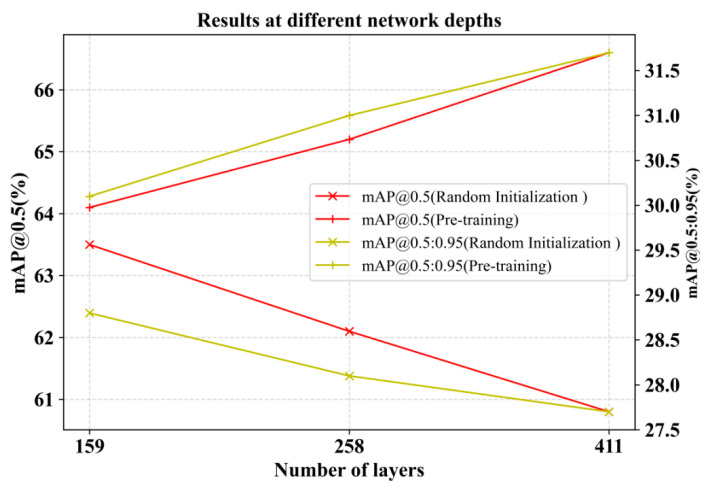
Results at different network depths. Comparing lines of the same color, pre-training is far superior to random initialization.

By deploying three backbone networks with different numbers of layers, ResNet18, ResNet50, and ResNet101, we explored the detection effect under both pre-training in Scheme II and random initialization conditions. A comparison of the results in Figure 5 shows that the detection accuracy after pre-training is substantially improved compared with random initialization. And pre-training is better compared to increasing the depth of the network. When the number of model layers is varied, changing the low-level information inherent in the image itself in the pre-training phase has a higher robustness than pre-training with random initialization. For small datasets like our self-made power line defect dataset, blindly increasing network depth may not necessarily have a good effect. An excessively deep training network can cause data overfitting, which is highly likely to cause the gradient to gradually decrease or even disappear. This leads to ineffective updates of the parameters of the shallow network, which hinders the optimization of training. So our proposed method of obtaining rich semantic information from shallow networks is very necessary.

3. Exploration of CSFM insertion locations

**Figure 6:**
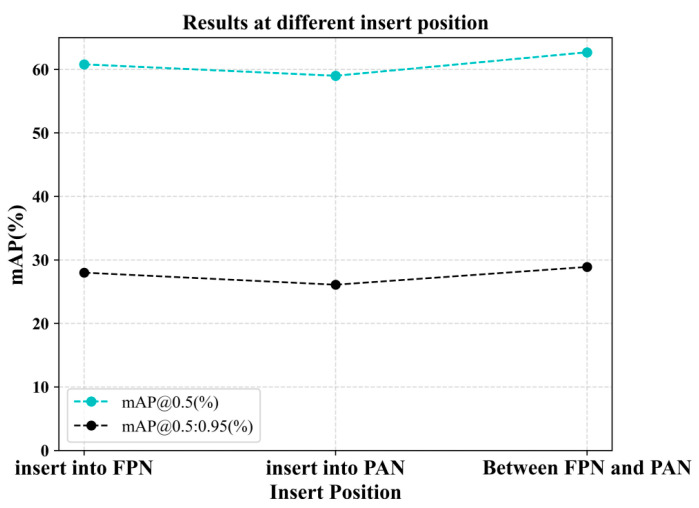
Results at different insert positions of CSFM. The experiments are conducted in 3 separate locations, inside the FPN, in the middle of the FPN and the PAN, and inside the PAN. Inserting CSFM between FPN and PAN works best.

The exploration of insertion locations is centered around the feature fusion part. This feature fusion network uses FPN with PAN structure, FAN with top-down sampling, and PAN with bottom-up sampling. The experiments are conducted in three separate locations, inside the FPN, in the middle of the FPN and the PAN, and inside the PAN. The experimental results are shown in Figure 6, and it can be seen that inserting in the middle of FPN and PAN has the best effect. So, we identified this insertion location. We found that both FPN and PAN are coherent structures used for feature fusion in a specific direction, and the order between them may have an impact on the feature representation. Inserting them internally can disrupt the coherence between feature layers. Inserting the CSFM between FPN and PAN may make it easier to capture key feature complementarity and coherence between the two structures, thereby improving detection accuracy. Overall, the choice of location for inserting the CSFM needs to be experimentally validated with the specific network architecture and task requirements. Insertion between FPN and PAN may make it easier to achieve effective feature fusion and improve detection accuracy. Insertion at other locations may be limited by factors such as feature representation, information transfer, and model complexity, leading to poor results. Therefore, these factors need to be considered in the model design, and careful experimentation and tuning should be performed to find the most suitable feature fusion strategy for a specific task.

## 5. Conclusions

In this paper, considering the limited computational resources of lightweight devices such as UAVs and the vital role of high-level semantic information for defect detection, we propose a strategy for extracting high-level semantic information using a lightweight network. The strategy consists of two parts. First, image segmentation and positional transformation are performed on large datasets to obtain an intrinsic semantic representation of the image, and transfer learning is utilized to enable this generalized semantic representation to guide the extraction of semantic features for shallow networks in new downstream tasks. Second, a category semantic fusion module is inserted into the feature fusion phase. This module supplements the global and local information to obtain more fusion of category semantic features on the basis that the channel attention can pay more attention to the category semantic information. After the combination of these two methods, rich semantic information can be extracted and fused without an excessive increase in network depth. We explored and debugged the usage strategy on the homemade power line dataset. Then, the most effective scheme was selected to successfully verify the effectiveness and generalization of the strategy on the publicly available insulator dataset SFID, Pascal VOC dataset.

## Figures and Tables

**Table 1:** Generic training parameters.

Training Parameters	Values
Batch size	64
Image size	448
Optimizer	SGD
Initial learning rate	0.01
Momentum	0.937
Weight decay	0.005
Focal loss gamma	0
Anchor-multiple threshold	4.0

**Table 2:** Confusion matrix.

	Ground Truth	Prediction
TP (True Positive)	positive	positive
TN (True Negative)	negative	negative
FP (False Positive)	positive	negative
FN (False Negative)	negative	positive

**Table 3:** Results of 16-block pre-training with CSFM on transmission power lines.

Pre-Training	Backbone	Neck	mAP@0.5
Random Initialization	ResNet18	FPN + PAN	63.5
Random Initialization	ResNet18	FPN + CSFM + PAN	64.1
**Pre-training _crop16d**	**ResNet18**	**FPN + CSFM + PAN**	**66.1**

**Table 4:** Results of 16-block pre-training with CSFM on Pascal VOC dataset.

Pre-Training	Backbone	Neck	mAP@0.5	mAP@0.5:0.95
Random Initialization	ResNet18	FPN + PAN	83.6	60.9
Random Initialization	ResNet18	FPN + CSFM + PAN	83.8	61.6
**crop16d**	**ResNet18**	**FPN + CSFM + PAN**	**84.5**	**62.5**

**Table 5:** Comparative experiments on synthetic fog insulator dataset for different networks.

Detection Mechanism	Detection Model	P	R	mAP@0.5	Number of Layers
FINet (SOTA)	FINet	93.1	99.5	99.5	311
Mainstream fault Detection mechanisms	Faster RCNN	-	-	98.4	-
	Mask RCNN	-	-	98.3	-
	YOLOX	-	-	99.4	-
	Swin-Transformer	-	-	99.0	-
	YOLOv5	-	-	99.3	266
**Ours**	**TL + CSFM**	**95**	**99.5**	**99.4**	**177**

TL: transfer learning, CSFM: category semantic fusion module.

## Data Availability

Public Pascal VOC dataset official website link: http://host.robots.ox.ac.uk/pascal/VOC/, accessed on 15 March 2023. The SFID and tutorials can be found on GitHub: https://github.com/zhangzhengde0225/FINet, accessed on 9 May 2023.
